# Evaluation of GABAergic Transmission Modulation as a Novel Functional Target for Management of Multiple Sclerosis: Exploring Inhibitory Effect of GABA on Glutamate-Mediated Excitotoxicity

**DOI:** 10.1155/2014/632376

**Published:** 2014-03-20

**Authors:** Ankit A. Gilani, Ranjeet Prasad Dash, Mehul N. Jivrajani, Sandeep Kumar Thakur, Manish Nivsarkar

**Affiliations:** ^1^Department of Pharmacology and Toxicology, National Institute of Pharmaceutical Education and Research, C/O-B. V. Patel Pharmaceutical Education and Research Development (PERD) Centre, S. G. Highway, Thaltej, Ahmedabad, Gujarat 380054, India; ^2^Department of Pharmacology and Toxicology, B. V. Patel Pharmaceutical Education and Research Development (PERD) Centre, S. G. Highway, Thaltej, Ahmedabad, Gujarat 380054, India

## Abstract

Multiple sclerosis (MS) is an autoimmune inflammatory disease of the central nervous system (CNS) where the communication ability of nerve cells in the brain and spinal cord with each other gets impaired. Some current findings suggest the role of glutamate excitotoxicity in the development and progression of MS. An excess release of glutamate leads to the activation of ionotropic and metabotropic receptors, thus resulting in accumulation of toxic cytoplasmic Ca^2+^ and cell death. However, it has been observed that gamma-aminobutyric acid-A (GABA_A_) receptors located in the nerve terminals activate presynaptic Ca^2+^/calmodulin-dependent signaling to inhibit depolarization-evoked Ca^2+^ influx and glutamate release from isolated nerve terminals, which suggest a potential implication of GABA_A_ receptor in management of MS. With this proof of concept, we tried to explore the potential of selective GABA_A_ receptor agonists or positive allosteric modulators (diazepam and phenobarbitone sodium) and GABA_A_ level enhancer (sodium valproate) for management of MS by screening them for their activity in experimental autoimmune encephalomyelitis (EAE) model in rats and cuprizone-induced demyelination model in mice. In this study, sodium valproate was found to show the best activity in the animal models whereas phenobarbitone sodium showed moderate activity. However, diazepam was found to be ineffective.

## 1. Introduction

Multiple sclerosis (MS) is an autoimmune inflammatory disease of the central nervous system (CNS) where the communication ability of nerve cells in the brain and spinal cord with each other gets impaired [1]. One of the major pathological conditions underlying the development of MS is demyelination of the axons [2]. Some current findings suggest the role of glutamate excitotoxicity in the development and progression of MS. Glutamate is the major excitatory neurotransmitter of the central nervous system, which has been proven to have a central role in a complex communication network established between all residential brain cells [3]. This has been proved from the magnetic resonance spectroscopy studies of MS brains that showed elevated glutamate levels in acute MS lesions [4]. An excess release of glutamate leads to the activation of ionotropic and metabotropic receptors, thus resulting in accumulation of toxic cytoplasmic Ca^2+^ and cell death. However, one interesting finding in this context is that GABA_A_ receptors located in the nerve terminals activate presynaptic Ca^2+^/calmodulin-dependent signaling to inhibit depolarization-evoked Ca^2+^ influx and glutamate release from isolated nerve terminals [5], which suggest a potential implication of GABA_A_ receptor in management of MS. Hence, preventing excitotoxic neuronal damage, by inhibition of glutamate release* via* GABAergic modulation, may serve as an effective approach for the treatment for MS.

This study was focused on evaluating the potential of selective GABA_A_ receptor agonists or positive allosteric modulators and GABA level enhancers for management of MS in experimental animal models. In particular, diazepam, phenobarbitone sodium, and sodium valproate were screened for their activity in EAE model induced in female* Wistar* rats and cuprizone-induced MS model in C57BL/6 mice. Both diazepam and phenobarbitone sodium are positive allosteric modulators of GABA_A_ whereas sodium valproate acts as GABA enhancer by inhibiting GABA transaminase that catabolizes GABA or blocks its reuptake into glia and nerve endings [6].

## 2. Materials and Methods

### 2.1. Materials

Diazepam, mitoxantrone HCl, and quetiapine fumarate were obtained as gift sample from Intas Pharmaceuticals, Ahmedabad, India. Sodium valproate was obtained as a gift sample from Chemclone Industries, Ahmedabad, India. Phenobarbitone sodium was purchased from Abbott India Ltd., Mumbai, India. Sodium chloride, potassium chloride, glucose, disodium hydrogen phosphate, potassium dihydrogen phosphate, hydrochloric acid, and glacial acetic acid were obtained from Qualigen Fine Chemicals, Mumbai, India. All the reagents and chemicals used for the study were of analytical grade. Paraformaldehyde, percoll, sucrose, disodium EDTA, Tris buffer, HEPES sodium salt, sodium bicarbonate, calcium chloride, magnesium chloride, 4-aminopyridine, glutamate, NAD^+^ (oxidized form), cresyl violet acetate stain, and luxol fast blue stain were purchased from Himedia Laboratories Pvt. Ltd., Mumbai, India. Cuprizone, Complete Freund's Adjuvant (CFA), and L-glutamic acid dehydrogenase solution (bovine liver origin) were obtained from Sigma Aldrich, USA. Isoflurane (Raman and Weil Pvt. Ltd., Daman, India) was used for anesthetizing animals. Heparin was purchased from Biological E. Ltd, Hyderabad, India. Deionised water for HPLC was prepared in-house using a Milli-Q integral water purification system (Millipore Elix, Germany).

### 2.2. Animals

Female* Wistar* rats weighing 150–200 g and male C57BL/6 mice weighing 25 to 30 gm were obtained from the animal house of B. V. Patel PERD Centre, Ahmedabad. Animal housing and handling were performed in accordance with Good Laboratory Practice (GLP) mentioned in CPCSEA guidelines. The animal house is registered with the Committee for the Purpose of Control and Supervision of Experiments on Animals, Ministry of Social Justice and Empowerment, Government of India, vide registration number 1661/PO/a/12/CPCSEA. All experimental protocols were reviewed and accepted by the Institutional Animal Ethics Committee prior to initiation of the experiment.

### 2.3. Experimental Autoimmune Encephalomyelitis (EAE) Model in Rats

#### 2.3.1. EAE Induction, Drug Treatment, and Clinical Scoring of the Symptoms

To induce EAE, spinal cord was isolated from female* Wistar* rats as previously described by Kennedy et al., 2011 [7]. However, transcardial perfusion of the rats was done according to the method described by Gage et al., 2012 [8], prior to isolation of the spinal cord. Following this, homogenate from the isolated rat spinal cord was prepared following the method reported by Shevach, 2001 [9], with certain modifications. Briefly, one gram of frozen rat spinal cord was homogenized at 13000 rpm on ice in 1 mL of 0.9% w/v NaCl (1 : 1 ratio) for 3 to 4 min. The homogenate was then emulsified in Complete Freund's Adjuvant by vortexing at high speed to give a final 1 : 2 (v/v) ratio of spinal cord to CFA (Note: it is very important to add the antigen to the adjuvant and not the other way round). The prepared final emulsion (50 *μ*L) was then injected into each hind footpad (subplantar region) of each rat in all the groups except normal control. The animals were observed daily for weight changes and clinical signs of EAE according to the following scale: 0 = no disease; 0.5 = distal limp tail; 1.0 = flaccid tail; 2 = mild paraparesis; 3 = moderate paraparesis; 3.5 = one hind limb is paralyzed; 4 = weakness of forelimbs with paraparesis or paraplegia and/or atonic bladder; 5 = complete hind limb paralysis; 6 = quadriplegia, moribund state, or death [10].

However, prior to immunization with spinal cord homogenate, animals were divided into 6 different groups of 6 animals each: group I, nonimmunized controls received normal saline; group II (disease control group), immunized and then received normal saline intraperitoneally (vehicle for all the treatments); group III (positive control), immunized and then received mitoxantrone HCl (dose: 0.5 mg/kg body weight intraperitoneally); group IV, immunized and then received diazepam (dose: 2.0 mg/kg body weight intraperitoneally); group V, immunized and then received phenobarbitone sodium (dose: 30.0 mg/kg body weight intraperitoneally), and group VI, immunized and then received sodium valproate (dose: 200.0 mg/kg body weight intraperitoneally). Mitoxantrone was used as a positive control in this experiment as it has been currently approved by FDA for the treatment of MS and has also been reported for reducing the signs and severity of EAE in animals [11]. The doses of diazepam, phenobarbitone sodium, and sodium valproate were the same as those used in some previous reports for their CNS-related activities in rats [12–14]. All the drugs were administered once a day for a period of 21 days.

On day 22, all the animals were euthanized using excess isoflurane anesthesia. This was followed by preparation of cerebrocortical synaptosomes by discontinuous percoll gradient method and glutamate release assay was performed [15].

#### 2.3.2. Preparation of Cerebrocortical Synaptosomes

The frontal cortex was isolated from all the animals and synaptosomes were purified on discontinuous percoll gradients as described previously [16]. Synaptosomes, which sediment between the 10% and 23% percoll bands, were collected and diluted in a final volume of 30 mL of HEPES buffer medium consisting of 140 mM NaCl, 5 mM KCl, 5 mM NaHCO_3_, 1 mM MgCl_2_, 1.2 mM Na_2_HPO_4_, 10 mM glucose, and 10 mM HEPES, pH 7.4. The resulting sample was then centrifuged at 27,000 ×g for 10 min at 4°C. The pellets formed were thus resuspended in 5 mL of HEPES buffer medium, and the protein content was determined using micro-BCA Protein Assay Reagent Kit (Pierce Cat #23235, Thermo Scientific, Rockford, IL, USA). Subsequently, the glutamate release assay was performed from the synaptosomal pellets. The pellets should be kept on ice and used within 3-4 h.

#### 2.3.3. Glutamate Release Assay

Glutamate release from cerebrocortical synaptosomes was determined using an assay in which exogenous glutamate dehydrogenase and NADP^+^ are used to couple the oxidative decarboxylation of the released glutamate. This results in the generation of NADPH which was then detected fluorimetrically [17]. Synaptosomal pellets were resuspended in HEPES buffer medium and incubated at 37°C for 3-4 min to facilitate polarization of the plasma membrane. Subsequently, 1 mM NADP^+^, 50 units/mL glutamate dehydrogenase, and 1.2 mM CaCl_2_ were added. The resulting sample was then incubated for 5 min, following which 3 mM 4-AP (4-amino pyridine) was added to stimulate the glutamate release. Fluorescence was then measured using spectrofluorometer (SL174, Systronics India Ltd.) at excitation and emission wavelengths of 359 nm and 468 nm, respectively. Data points were obtained at 10 sec intervals. 4-AP (potassium channel blocker) was used in this study to destabilize the plasma membrane potential of the synaptosomes, which subsequently results in an increase in the cytoplasmic free Ca^2+^ concentration through the opening of voltage-gated Ca^2+^ channels. Finally, this leads to generation of spontaneous action potentials which are capable of triggering the exocytotic release of glutamate [15].

### 2.4. Cuprizone-Induced Demyelination in Mice

#### 2.4.1. Model Development and Treatment

Thirty-six male C57BL/6 mice (*n* = 6) were used in the study. Experimental group, route of administration, and treatment protocol were the same as those of EAE model. However, the doses of the drugs were as follows: diazepam, 1.0 mg/kg body weight; phenobarbitone, 20.0 mg/kg body weight; and sodium valproate, 150.0 mg/kg body weight. The doses of diazepam, phenobarbitone sodium, and sodium valproate were the same as those used in some previous reports for their CNS-related activities in mice [13, 18, 19]. Acute demyelination was induced by feeding the animals a diet containing 0.2% cuprizone (bis(cyclohexanone)oxaldihydrazone) mixed into a ground standard rodent chow* ad libitum* for a period of 4 weeks. The food was freshly prepared as cuprizone is an unstable compound. Control animals received normal pellet diet. Diazepam, phenobarbitone sodium, and sodium valproate were administered daily for 28 days starting from the same day as of cuprizone treatment. Quetiapine fumarate (dose: 10 mg/kg bodyweight) was used as a positive control since it has been reported that chronic administration of quetiapine attenuates myelin breakdown in the cerebral cortex of cuprizone-exposed mice [20]. All the drugs were administered once a day for a period of 28 days.

#### 2.4.2. Tissue Preparation, Staining, and Microscopy

On day 29, tissue evaluation was performed. All the animals were anaesthetized using isoflurane anaesthesia and perfused transcardially with 10 U/mL heparin in phosphate buffer saline (pH 7.4) followed by 4% w/v paraformaldehyde. The brains were isolated and 5 *μ*m sectioning of the coronal sections was done using a rotary microtome (Model 0126, Yorco, India). The slides were then stained with luxol fast blue and incubated overnight at 60°C followed by secondary staining with cresyl violet acetate for 5 min. Tissue sections were observed for all possible changes in the anatomy under Olympus IX51 optical microscope (Olympus Optical Co. Ltd., Tokyo, Japan). The photographs were taken with an Olympus TL4 camera. The observations were compared to detect the extent of demyelination.

## 3. Results

### 3.1. Clinical Scoring of the Symptoms in EAE Model

**Figure 1:**
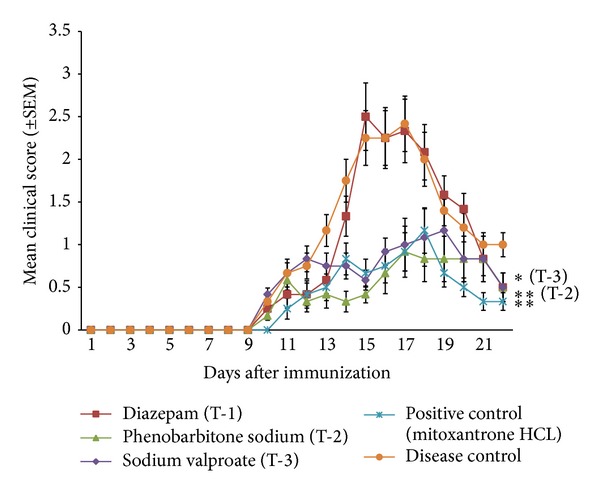
Changes in neurological/clinical signs of EAE with days after immunization. Significant difference was found in the mean clinical scores (±SEM), [*n* = 6] of animals of phenobarbitone (T-2) and sodium valproate (T-3) groups as compared to the diseased control group, [*P ≤ 0.05 by one way ANOVA (Dunnett's multiple comparisons test)].

The clinical symptoms of EAE in different animal groups are shown in Figure 1. The clinical scores for the phenobarbitone sodium and sodium valproate treated animals were significantly less (*P* ≤ 0.05) as compared to the diseased group animals. Moreover, the inhibition pattern of clinical symptoms of the above mentioned treatment groups was similar to positive control group. However, no significant inhibition of EAE symptoms was found in the diazepam treated animals as compared to the disease control group, indicating that it may not be effective in preventing the disease progression.

### 3.2. Glutamate Release Assay in EAE Model

**Figure 2:**
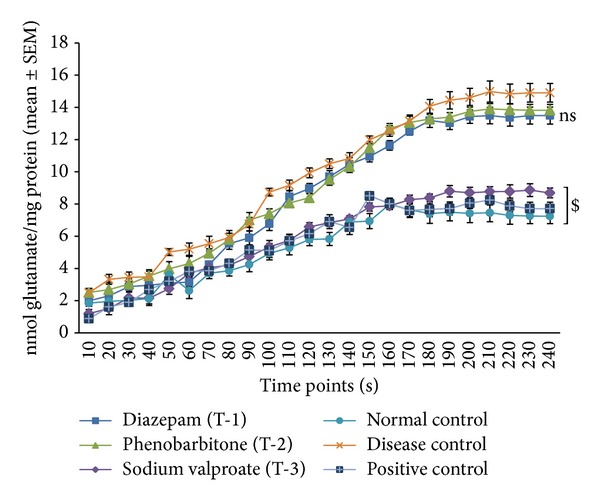
Glutamate released from the synaptosomes at different time points detected fluorimetrically. Diazepam and phenobarbitone did not show any inhibition in the glutamate release. Glutamate release from the synaptosomes of the animals of sodium valproate group (T-3) group was significantly lower than the diseased control group [*n* = 6], [*P ≤ 0.05 by one way ANOVA (Dunnett's multiple comparisons test)]. * indicates that the observed neurological/clinical signs of EAE in animals of T-2 and T-3 group were significantly different (*P* ≤ 0.05) from that of disease control animals.

The amount of glutamate released (nmol/mg protein) at different time intervals from the synaptosomes of animals of all the groups is shown in Figure 2. The results showed an excessive release of glutamate from the synaptosomes of the diseased control group (indicating the occurrence of glutamate excitotoxicity) whereas the levels of glutamate released from the synaptosomes of the animals of normal control and positive control groups were significantly low (*P* ≤ 0.05). However, no significant difference was observed in the glutamate release profiles of the animals treated with diazepam and phenobarbitone when compared to the diseased control group. This indicates that they did not produce any inhibition of glutamate release. Sodium valproate treated animals showed a glutamate release profile much similar to that of normal and positive control groups and was also significantly different (*P* ≤ 0.05) as compared to the diseased animals.

### 3.3. Histological Observation in Cuprizone-Induced Demyelination in Mice

**Figure 3:**
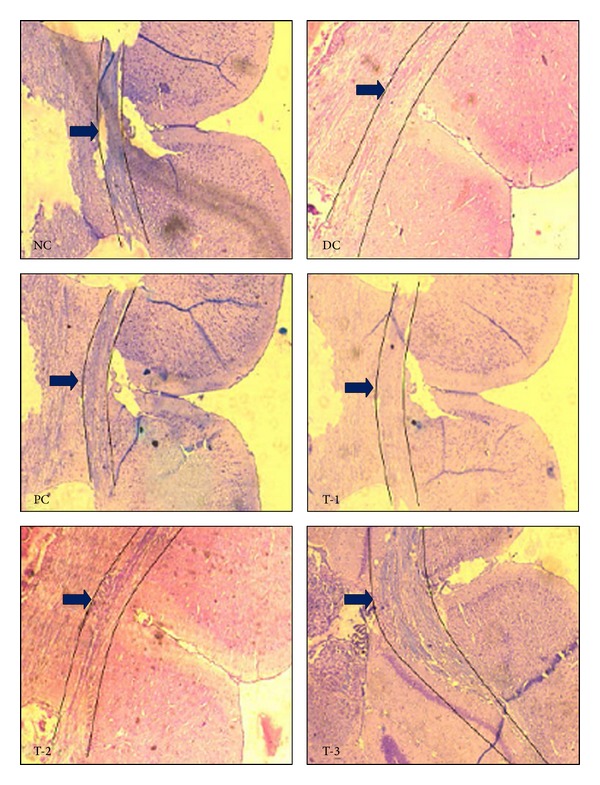
Luxol fast blue stained coronal sections of C57BL/6 mice brains observed under 40x magnification. The region of corpus callosum is highlighted and indicated by the arrow. Blue colour indicates presence of myelin. (NC: normal control; DC: disease control; PC: positive control; T-1: diazepam treatment; T-2: phenobarbitone sodium treatment; T-3: sodium valproate treatment.)

Luxol fast blue stained coronal sections observed under 40x magnification are shown in Figure 3 where myelinated axons were stained blue while nonmyelinated axons were stained pink. The confirmation of successful model development was confirmed from prominent demyelination in the region of corpus callosum of diseased control group. However, tissue sections of normal and positive control animals showed blue colored bands which indicate the presence of myelinated axons. In this experiment, it was observed that sodium valproate inhibited demyelination, as majority of the axons in corpus callosum stained blue while few others stained pink. However, phenobarbitone showed moderate protection against cuprizone-induced demyelination (thin and less intense bands of blue colored myelinated fibers in the sections). The images revealed a prompt demyelination in the diazepam treated animals, thus indicating that diazepam was not able to inhibit demyelination induced by cuprizone.

## 4. Discussion

Previous studies have shown that the concentration of GABA and the glutamate decarboxylase activity in blood are reduced in EAE and MS [21, 22]. Based on these findings, an attempt was made to explore the potential of selective GABA_A_ receptor agonists or positive allosteric modulators and GABA level enhancers for treatment of MS. In the present study, diazepam, phenobarbitone sodium, and sodium valproate were screened for their activity against MS in EAE model in rats and cuprizone-induced demyelination model in mice. In context to the EAE model, it was observed that phenobarbitone sodium and sodium valproate inhibited the clinical symptoms of EAE; however, diazepam failed to control these EAE symptoms. Although both diazepam and phenobarbitone are positive allosteric modulators of the GABA_A_ receptors, the probable reason for the difference in their observed activity may be the difference in their binding sites [23]. Phenobarbitone sodium and diazepam acts independently, by binding to the beta subunit and benzodiazepine binding site of GABA_A_ receptor, respectively. Diazepam has only GABA facilitatory action whereas phenobarbitone has GABA facilitatory as well as GABA mimetic effect [23]. This means that it not only enhances the action of GABA but also causes opening of the Cl^−^ ion channels. Moreover, phenobarbitone also blocks calcium channels, thus resulting in inhibition of excitatory transmitter release. Sodium valproate is an inhibitor of GABA transaminase and increases GABA levels which may be responsible for inhibition of glutamate excitotoxicity and hence demyelination in EAE animals.

However, in context to the glutamate release assay, sodium valproate was found to be the most effective amongst all the three drugs. It significantly inhibited both clinical signs and glutamate release which may be attributed to its ability to enhance the inhibitory effect of GABA on glutamate release and thus preventing glutamate excitotoxicity. It was observed that although phenobarbitone inhibited the clinical signs of EAE, it failed to inhibit glutamate release. Although the reason for this finding is still unclear, it may be presumed that some other pathway is involved in its mechanism of action.

In the cuprizone-induced demyelination in C57BL/6 mice, it was observed that diazepam did not produce any inhibition of demyelination while sodium valproate inhibited demyelination to good extent which was comparable with that of positive control. The animals treated with phenobarbitone sodium showed moderate inhibition of demyelination. Cuprizone induces alterations of mitochondrial morphology and it is also speculated that the neurotoxic properties of this copper-chelating compound are due to a disturbance of cellular respiration which is a key function of mitochondria. Moreover, it appears that the activity of a set of enzymes is disturbed prior to myelin loss during the first days or weeks of cuprizone exposure [24]. However, no reports are available with respect to the activity of sodium valproate and phenobarbitone sodium on cellular response of mitochondria. Thus, further mechanistic studies are required to understand the exact reason underlying these findings and elucidate the pathway by which these drugs showed their pharmacological activity in this model.

## 5. Conclusion

The findings of this study indicate that sodium valproate may serve as a potential drug for management of MS. However, this is only a preliminary finding and further extensive studies are required in this context. Moreover, new drug candidates selectively acting by GABAergic system may be designed and screened for their activity against MS.
